# Nanocellulose-Linked MXene/Polyaniline Aerogel Films for Flexible Supercapacitors

**DOI:** 10.3390/gels8120798

**Published:** 2022-12-05

**Authors:** Liying Xu, Wenxuan Wang, Yu Liu, Daxin Liang

**Affiliations:** 1 School of Food Engineering, Harbin University, Harbin 150086, China; 2 Key Laboratory of Bio-Based Material Science and Technology (Ministry of Education), Northeast Forestry University, Harbin 150040, China

**Keywords:** supercapacitors, MXene, CNFs, polyaniline

## Abstract

In the development of energy supply systems for smart wearable devices, supercapacitors stand out owing to their ability of quick and efficient energy supply. However, their application is limited due to their low energy density and poor mechanical energy. Herein, a strategy for the preparation of flexible supercapacitors is reported, which is based on the fabrication of aerogel films by simultaneously utilising cellulose nanofiber (CNFs) as an MXene intercalation material and polyaniline (PANI) as a template material. CNFs, which can form hydrogen-bonded networks, enhance the mechanical properties of MXene from 44.25 to 119.56 MPa, and the high electron transport properties of PANI endow MXene with a capacitance of 327 F g^−1^ and a resistance of 0.23 Ω. Furthermore, the combination of CNFs and PANI enables a 71.6% capacitance retention after 3000 charge/discharge and 500 folding cycles. This work provides a new platform for the development of flexible supercapacitors.

## 1. Introduction

As an alternative to fossil energy, electric power is a renewable and clean energy that is easy to store and transport [1]. For daily life applications, structural electrode materials with high flexibility and high electrochemical performance attracted have great attraction [2,3,4], because they are essential for manufacturing lightweight and flexible electronic products [5,6,7]. In particular, capacitors offer fast charging and discharging speed and good recyclability for practical applications [8]. To fabricate flexible capacitors, carbon-based materials, including carbon nanotubes and graphene, have been widely used as electrode materials with stable performance and structure [9,10,11]. Unfortunately, their electrochemical energy storage capacity is insufficient [12]. Although this problem can be significantly circumvented by adding metal oxides and conductive polymers, the reduction of the mechanical properties stemming from the lack of interaction between the two materials cannot be ignored [13,14].

MXenes (Ti_3_AlC_x_) are a family of two–dimensional (2D) transition metal carbides or nitrides having the general formula M_n+1_X_n_TX (n = 1–3), where M represents an early transition metal such as Ti, Zr, V, Nb, Ta or Mo, X is the carbon or nitrogen element and T represents a surface functional group, generally =O, –OH or –F [12,15]. MXenes are prepared by selectively etching the A element layer from the corresponding MAX phase precursor (M_n+1_AX_n_) [16]. Since Gogotsi et al. synthesised the first MXene in 2011 [17,18], these materials have attracted a wide interest for application in supercapacitors. Their unique 2D layered structure and rich surface functional groups provide them with excellent conductivity and high surface hydrophilicity. However, their mechanical properties are still not ideal [19,20].

Cellulose nanofibrils (CNFs) have useful properties, such as strong mechanical strength, distinctive biological flexibility, great stability and outstanding capacity of liquid absorption [21,22,23]. Moreover, as a nanolinear structured material, CNFs can not only enhance the mechanical properties of MXene by dispersing between its interlayer gaps but also improve the electrochemical performance by inhibiting the stacking of MXene and facilitating the transport of electrolyte ions as an electrolyte reservoir [24,25,26]. However, the charge storage capacity of MXene in supercapacitors stems mainly from =O functional groups, whereas –F and –OH functional groups, which are abundant in CNFs, are detrimental to the electrical performance [12,27].

Polyaniline (PANI) is a typical conducting polymer that has been widely used to prepare supercapacitor electrodes due to its fast redox rate, high pseudocapacitance, fully reversible doping, low cost and easy synthesis [28,29,30]. However, the application of PANI is limited by its tendency to undergo agglomeration, which leads to the blockage of the conductive paths, increase in resistance and decrease in energy density decreases [31,32,33]. Interestingly, PANI can be uniformly dispersed using CNFs as templates and excess –OH as linkages, thereby leading to the improvement of their electrochemical performance.

**Scheme 1:**
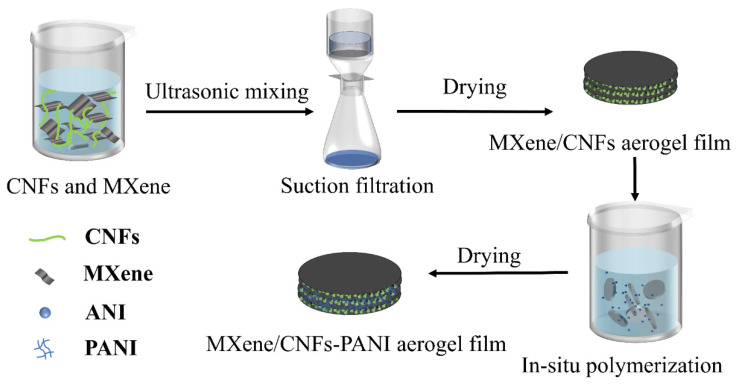
Preparation of MXene/CNF aerogel films and MXene/CNF–PANI aerogel films.

Herein, MXene/CNF–PANI aerogel films with layered porous structures were prepared via in situ polymerisation and using MXene/CNF aerogel films. The films were obtained by self-assembling MXene and CNFs via vacuum suction filtration after preparing single-layer MXene suspensions by LiF/HCl etching of Ti_3_AlC_2_ (Scheme 1). CNFs served as an intercalation material to link MXene with PANI, which greatly enhanced the mechanical properties of the MXene films, and as a template material to enable PANI to disperse. Moreover, PANI provided a communicating pathway between MXene layers and improved the electrochemical properties of materials, demonstrating a new structural model for the preparation of flexible supercapacitors for daily life applications.

## 2. Results and Discussion

### 2.1. Characterisation of MXene/CNF Aerogel Films and MXene/CNF–PANI Aerogel Films

#### 2.1.1. Scanning Electron Microscopy (SEM) Analysis

**Figure 1:**
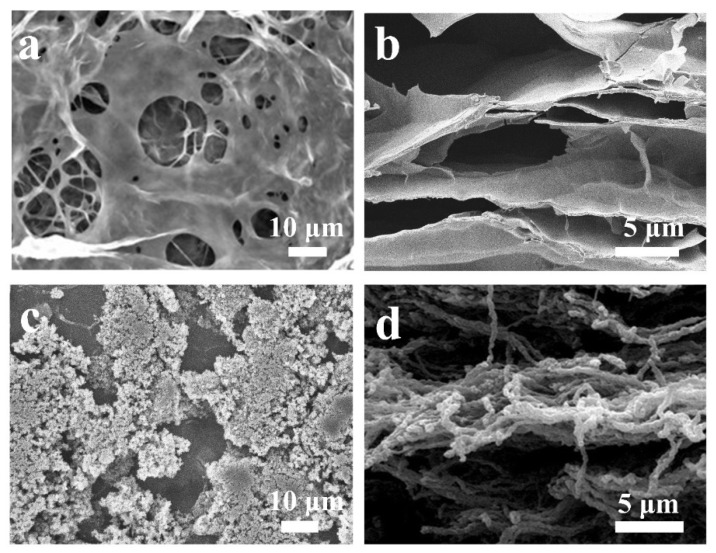
SEM images of (**a**) the surface; (**b**) cross section of MXene/CNF aerogel films; (**c**) the surface; (**d**) cross section of MXene/CNF–PANI aerogel films.

The micro-morphologies of the MXene/CNF and MXene/CNF–PANI aerogel films are shown in Figure 1. Due to the insertion of CNFs between the MXene layers having a rough surface (Figure 1a), the MXene/CNF aerogel films were endowed with a layered and porous structure (Figure 1b), which is conducive to the infiltration of electrolytes. Moreover, this uniform dispersion can be expected to enhance the mechanical properties of MXenes. The polymerisation of PANI on the CNFs transformed the smooth surface (Figure 1c) to an overall layered porous structure (Figure 1d), illustrating the main roles of PANI, which were to coat the CNFs and connect the MXenes that were disconnected after the incorporation of the CNFs for an improved electrochemical performance.

#### 2.1.2. Fourier Transform Infrared (FTIR) Analysis

**Figure 2:**
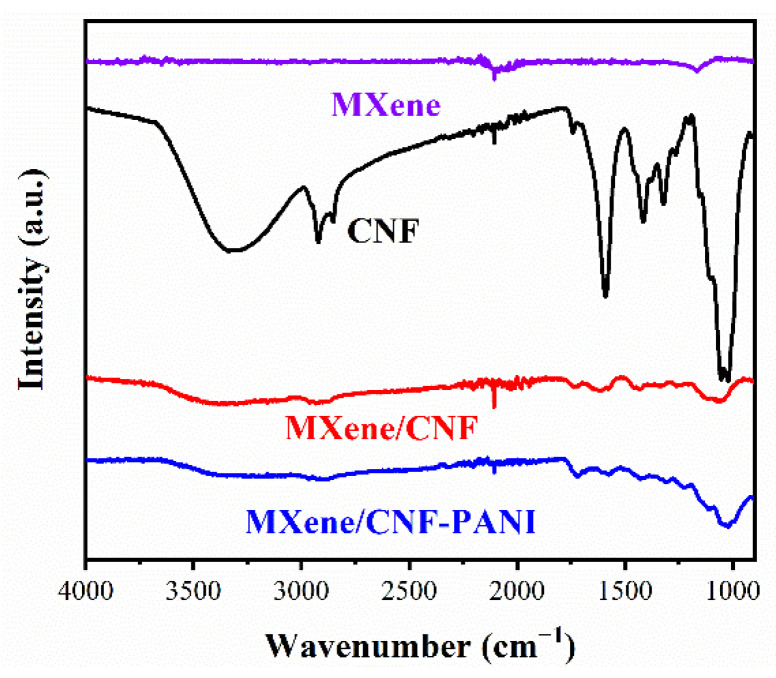
FTIR spectra of CNFs, MXene, MXene/CNF aerogel films and MXene/CNF–PANI aerogel films.

The FTIR spectra of CNFs, MXene, MXene/CNF aerogel films and MXene/CNF–PANI aerogel films are shown in Figure 2. In the spectrum of CNFs, the peaks at 3344, 2932–2868 and 1031 cm^−1^ can be assigned to the stretching vibration of –OH, the stretching vibrations of –CH and the C–O–C pyranose ring skeleton of CNFs, respectively. The spectrum of MXene shows a characteristic peak at 1100 cm^−1^ corresponding to the surface C–F end groups. [34]. When CNFs were added into MXene, the absorption peaks of C–F were masked and the intensity of the –OH peaks increased because a large amount of –OH groups was introduced, whereas the spectrum of the MXene/CNF–PANI aerogel films only exhibited stretching vibrations of N–H between 3350–3344 cm^−1^ and double-bond vibrations of benzene and quinone ring molecules of the PANI chains at 1486 and 1579 cm^−1^, respectively [35]. This result demonstrates that CNFs serve only as a structural framework to construct the MXene/CNF–PANI aerogel films, in which PANI connects the MXenes by reducing the –OH on the surface of the CNFs, thereby improving the electrochemical performance of the MXene/CNF–PANI aerogel films.

#### 2.1.3. X-ray Diffraction (XRD) Analysis

**Figure 3:**
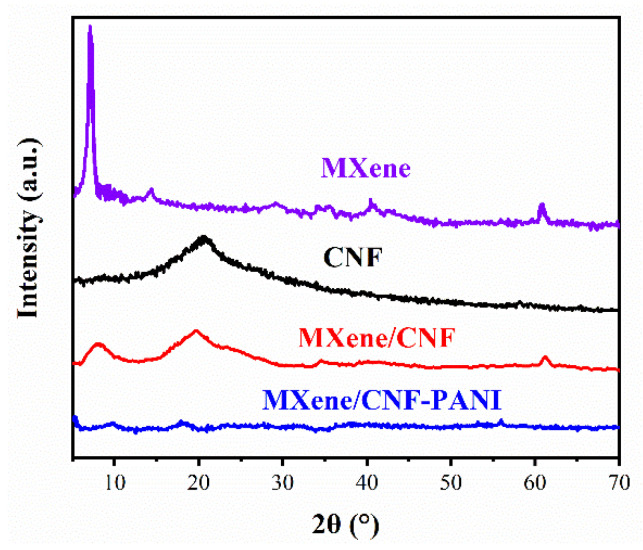
XRD patterns of CNFs, MXene, MXene/CNF aerogel films and MXene/CNF–PANI aerogel films.

Figure 3 shows the XRD patterns of CNFs, MXene, MXene/CNF aerogel films and MXene/CNF–PANI aerogel films. A distinct (002) diffraction peak was observed for MXene at 2θ = 7.1° [36], but it decreased with the addition of CNFs, indicating that the interlayer spacing of the MXene increased as a result of the successful penetration of cellulose between those layers. After the polymerisation of PANI, the diffraction peaks of the CNFs disappeared, which is in agreement with the SEM results, indicating that PANI coated the surface of cellulose.

#### 2.1.4. Tensile Stress–Strain Analysis

**Figure 4:**
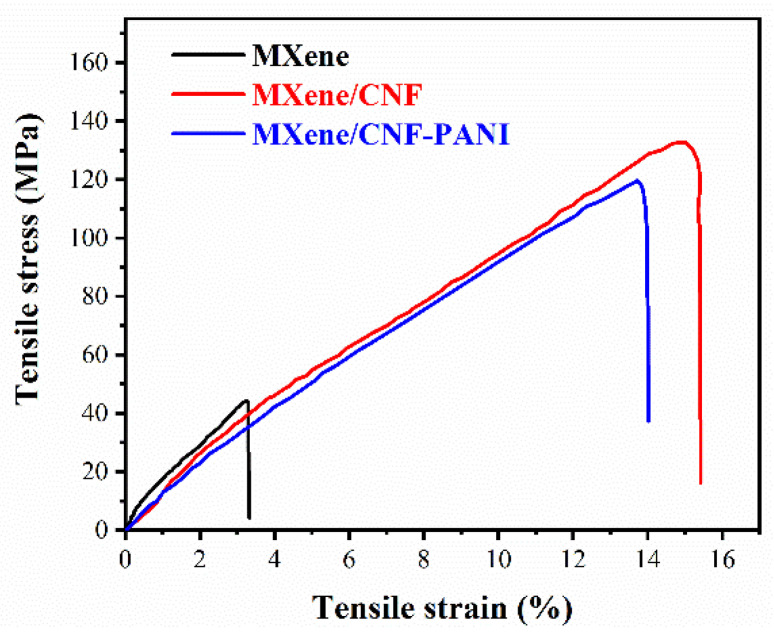
Tensile stress–strain curves of MXene, MXene/CNF aerogel films and MXene/CNF–PANI aerogel films.

Figure 4 shows the tensile stress–strain curves of CNFs, MXene, MXene/CNF aerogel films and MXene/CNF–PANI aerogel films. After the introduction of CNFs, which resulted in a uniform dispersion of stress in the MXene film, the stress strength of the MXene/CNF aerogel films increased from 44.25 to 132.84 MPa and their strain increased from 3.26% to 15.01% due to the hydrogen bonding interactions between the CNF networks. However, when PANI was introduced, it covered the surface of the CNFs and decreased the number of hydrogen bonds, reducing the stress strength and the strain of the MXene/CNF–PANI aerogel films, albeit only slightly, from 132.84 to 119.56 MPa and from 15.01% to 13.71%, respectively.

### 2.2. Electrochemical Performance of MXene/CNF Aerogel Films and MXene/CNF–PANI Aerogel Films

**Figure 5:**
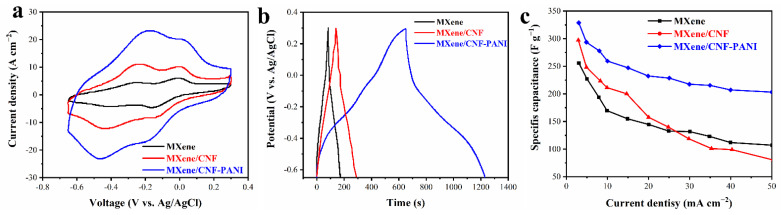
(**a**) CV curves, (**b**) GCD curves and (**c**) specific capacitance of MXene films, MXene/CNF aerogel films and MXene/CNF–PANI aerogel films.

As shown in Figure 5a, the cyclic voltammetry (CV) curve of the MXene films recorded at a scan rate of 2 mV s^−1^ exhibited a rectangular shape, which is indicative of their ideal capacitive behaviour. Upon addition of CNFs and PANI, an obvious increase in the peak current density in the CV curves at the same scan rate was observed. The results of a galvanostat charge/discharge (GCD) test were consistent with those of the CV measurements (Figure 5b). The GCD curves at the same discharge current density (3 mA cm^−2^) further demonstrate the higher mass capacitance of the MXene/CNF aerogel films; the insertion of CNFs endowed the MXene with a more free interlayer space for charge storage while retaining the interlayer electron transport channels for high interlayer conductivity. Two pairs of broad redox peaks were clearly observed in the potential window of the MXene/CNF aerogel films after the addition of PANI, indicating that the capacitance is mainly a pseudocapacitance because the reversible intercalation/deintercalation of protons leads to a valence state change of the redox element Ti. The GCD curves exhibited a distorted triangle due to the redox reaction of MXene, which was consistent with the CV results. The mass capacitance results calculated from the GCD curves are shown in Figure 5c. After the introduction of CNFs, the capacitance of the MXene/CNF aerogel films reached 294 F g^−1^, whereas pure MXene produced only a capacitance of 271 F g^−1^ at the same scan rate. When the scan rate was increased to 10 mV s^−1^, the MXene/CNF aerogel films still maintained a capacitance of 227 F g^−1^ and a capacitance ratio of 77%, which was much higher than that of the MXene films with a capacitance of 132 F g^−1^ (48%). The results showed that the improvement of the capacitive properties of the MXene/CNF aerogel films was related to the electrode structure, and the CNF nanonetwork structure not only prevented the stacking of MXene but also accelerated the intercalation/extraction transport rate of ions and improved the capacitance of MXene [20]. After PANI loading, the capacitance of the resulting aerogel films reached 327 F g^−1^ with a ratio of 77% when the scan rate was increased to 10 mV s^−1^. This large improvement was due to the fact that the introduction of PANI in the MXene/CNF aerogel films resulted in the formation of the loops inside, allowing a smooth electron transport.

**Figure 6:**
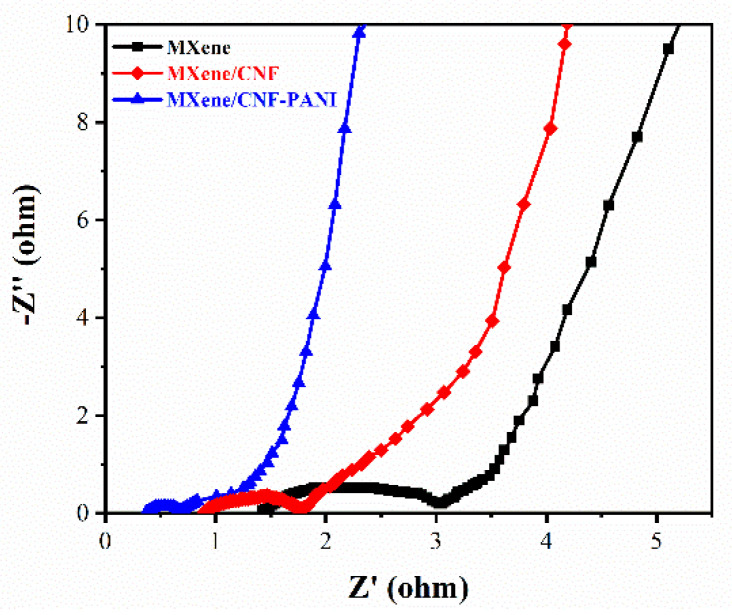
Nyquist curves of MXene films, MXene/CNF aerogel films and MXene/CNF–PANI aerogel films.

To understand the kinetic process of the charge/discharge profiles of the films, electrochemical impedance spectroscopy (EIS) measurements were conducted. Figure 6 shows the Nyquist curves of CNFs, MXene, MXene/CNF aerogel films and MXene/CNF–PANI aerogel films, in which two regions can be observed, i.e., semicircular arcs at high frequencies and a straight line at low frequencies [34,37,38,39]. For the MXene/CNF and MXene/CNF–PANI aerogel films, the straight-line regions are almost parallel to the Z′′ axis, indicating that ions could diffuse rapidly from the electrolyte solution to the film interface. MXene exhibited a relatively high resistance (2.5 Ω) due to the stacking of MXene nanosheets in a bi-continuous structure. In contrast, the addition of CNFs improved the stacking in the MXene/CNF aerogel films, reducing the internal resistance to 2.15 Ω. Then, the internal pathway constructed through the action of PANI was beneficial for a smooth electron transport, and the resistance value was reduced to 0.23 Ω, which proved that the introduction of MXene/CNF with PANI was effective.

**Figure 7:**
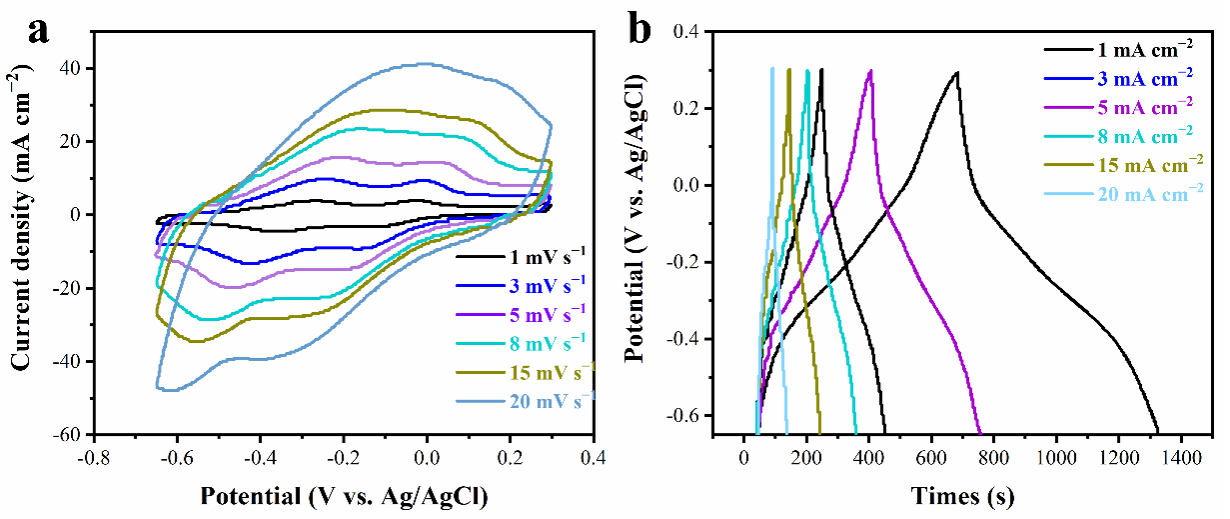
(**a**) CV curves and (**b**) GCD curves.

To demonstrate the effectiveness of this structural design for aerogel films, CV curves of the MXene/CNF–PANI aerogel films were recorded at varying scan rates from 1 to 20 mV s^−1^ (Figure 7a). At low scan rates, redox peaks appeared in the CV curves, which might be attributed to the special morphological features of the MXene/CNF–PANI aerogel films facilitating the protonation of the oxygen functional groups at positive and negative potential sites. As shown in Figure 7b, the charge/discharge profiles in a symmetric state reflect the nearly 100% Coulombic efficiency of the MXene/CNF–PANI aerogel films, indicating the occurrence of reversible redox reactions in the electrode. According to the results calculated from the GCD curves (Figure 5c), the MXene/CNF–PANI aerogel films showed high capacitive properties, i.e., 327 F g^−1^ at 3 mA cm^−2^ and 203 F g^−1^ at 50 mA cm^−2^, indicating that the MXene/CNF–PANI aerogel films exhibited good ion transport ability and that the three-dimensional structure design of MXene was useful and efficient.

**Figure 8:**
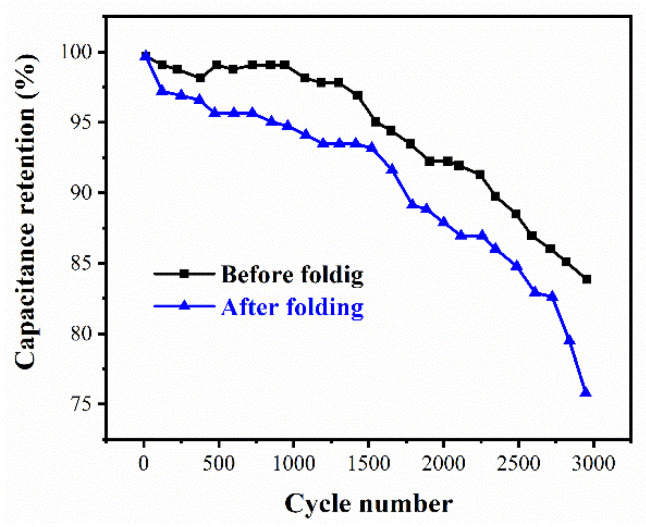
Capacitance of MXene/CNF–PANI aerogel films before and after 500 folding cycles and 3000 charge/discharge cycles at a constant charge/discharge current density.

To evaluate the applicability of the MXene/CNF–PANI aerogel films, 3000 charge/discharge cycles were performed at a current density of 50 mA cm^−2^ (Figure 8). The combination of CNFs and PANI prevented the stacking of MXene and increased the film toughness, whereas the resistance was not increased, resulting in a capacitance retention of 84.1% after 3000 charge/discharge cycles. Furthermore, a capacitance retention of 71.6% was obtained after subjecting the MXene/CNF–PANI aerogel films to 500 folding cycles at the same charge/discharge current density, indicating that the electrode material had excellent flexibility for practical applications [40]. The comprehensive performance of MXene/CNF–PANI aerogel films could be shown through Table 1, and the applicability of MXene/CNF–PANI aerogel films was shown through comparison.

## 3. Conclusions

In this study, porous layered MXene/CNF–PANI aerogel films with a novel structure were prepared, which can be used as supercapacitors. Using PANI for cladding after intercalation of CNFs into the MXene layers boosted the performance of MXene in terms of the three following properties: (1) The MXene/CNF–PANI aerogel films possessed a capacitance of 327 F g^−1^ and a resistance of 0.23 Ω because the introduction of CNFs prevented the stacking of MXene, enabling more free interlayer space for charge storage. Meanwhile, the introduction of PANI provided communicating pathways for a smooth electron transport. (2) A high mechanical strength of 119.56 MPa originating from the introduction of CNFs resulted in the formation of abundant hydrogen bonds to enhance the overall MXene structure. (3) The MXene/CNF–PANI aerogel films showed a capacitance retention of 71.6% after 500 folding and 3000 charge/discharge cycles. In summary, the MXene/CNF–PANI aerogel films are promising as a supercapacitor material with enhanced electrochemical, mechanical and cycling properties.

## 4. Materials and Methods

### 4.1. Materials

All chemicals were used without further purification. Titanium aluminium carbide (Ti_3_AlC_2_, ~400 mesh, 98%) was purchased from Yiyi Technology Co., Ltd., Jilin, China, CNF suspension (0.55 wt%) from Hongqi Technology Co., Ltd., Guilin, China and sulphuric acid (H_2_SO_4_, 99.8%) from Sinopharm Chemical Reagent Co., Ltd., Shanghai, China). Lithium fluoride (LiF, 99.7%), ammonium persulfate ((NH_4_)_2_S_2_O_8_, 99.7%), aniline (C_6_H_7_N, 97.7%) and potassium hydroxide (KOH, 97.7%) were obtained from Sigma-Aldrich Co., Ltd., Shanghai China. Hydrochloric acid (HCl, 37%) and ethanol (C_2_H_6_O, 99%) were sourced from a local supplier.

### 4.2. Characterisation

The morphology and structural characteristics of the samples were observed via SEM using a TM3030 microscope (Hitachi, Tokyo, Japan). The crystal structures were analysed using a D/max-2200VPC X-ray diffractometer (Rigaku, Tokyo, Japan). FTIR spectroscopy (Perkin Elmer, Waltham, MA, USA) was used to characterise the functional groups in the samples. A universal testing machine (CMT5504, MTS, Eden Prairie, MN, USA) was used to study the mechanical properties. All electrochemical performance tests were conducted on an electrochemical workstation (51-XMX1004, Oxford, UK).

### 4.3. Preparation of MXene, MXene/CNF Aerogel Films and MXene/CNF-PANI Aerogel Films

To an aqueous solution of LiF (6 g) and 9 M HCl, 5 g Ti_3_AlC_2_ was slowly added under continuous stirring at 45 °C for 24 h. After centrifugation of the solution, the resulting precipitate was washed first with HCl (1 M) for 2–3 times and then with deionised water until pH 7. After sonication for 60 min at 0 °C in water and centrifugation at 3500 rpm min^−1^ for 10 min, a dark green supernatant was obtained, which was collected and subjected to freeze-drying to furnish layered MXene flakes.

CNF films and MXene films were obtained by suction filtration from CNF and MXene solutions, respectively. The MXene/CNF aerogel films were prepared via insertion and suction filtration by blending 3.6 mL CNF solution (0.55 wt%) with 6.6 mL MXene solution (3 mg mL^−1^), followed by magnetic stirring at 500 rpm for 3 h, sonication for 30 min and then vacuum suction. The MXene/CNF aerogel films were immersed in 20 mL (NH_4_)_2_S_2_O_8_ solution (0.57 g mL^−1^) containing 1 M HCl solution for 3 h and then removed and immersed in 20 mL aniline solution (0.19 g mL^−1^) with 1 M HCl solution for 3 h for in situ polymerisation of aniline. MXene/CNF–PANI films were finally obtained by repeated rinsing with deionised water and drying.

### 4.4. Electrochemical Performance of MXene, MXene/CNFs Aerogel Films and MXene/CNFs-PANI Aerogel Films

The electrochemical performance of MXene, MXene/CNF aerogel films and MXene/CNF–PANI aerogel films was investigated by means of CV, GCD and EIS measurements using an electrochemical workstation. The composite films were cropped into 2 × 1.8 cm^2^ electrode sheets and assembled into symmetric supercapacitors in the electrochemical workstation using a three electrodes system with Ag/AgCl as the reference electrode and 3 M H_2_SO_4_ as the electrolyte.

## Figures and Tables

**Table 1:** MXene/CNF–PANI aerogel films comparison of the performance of capacitors with other capacitors.

Composites	Capacitance	Mechanical Properties (MPa)	Resistance (Ω)	Ref.
TEMPO-oxidized-nanocellulose carbon nanotubes	65 F g^−1^	0.065	26	[41]
N-doped porous carbon	193 F g^−1^	---	0.97	[42]
Ti_3_C_2_T_x_/CNF	298 F g^−1^	0.004	0.003	[12]
MWNT/cellulose nanofibers	145 F g^−1^	---	0.37	[43]
Brazilian-pine fruit coat	0.87 F cm^−2^	6.1	13.5	[39]
MXene/CNF–PANI	327 F g^−1^	119.56	0.23	This study

## Data Availability

Not applicable.
